# Phylogeny of *Salmonella enterica* subspecies *arizonae* by whole-genome sequencing reveals high incidence of polyphyly and low phase 1 H antigen variability

**DOI:** 10.1099/mgen.0.000522

**Published:** 2021-02-04

**Authors:** Nikki W. Shariat, Ruth E. Timme, Abigail T. Walters

**Affiliations:** ^1^​ Department of Population Health, College of Veterinary Medicine, University of Georgia, Athens, Georgia, USA; ^2^​ Center for Food Safety and Applied Nutrition, US Food and Drug Administration, College Park, Maryland, USA

**Keywords:** H antigen, polyphyly, *Salmonella*, subspecies *arizonae*, whole-genome sequencing

## Abstract

*
Salmonella enterica* subspecies *arizonae* is frequently associated with animal reservoirs, particularly reptiles, and can cause illness in some mammals, including humans. Using whole-genome sequencing data, core genome phylogenetic analyses were performed using 112 *S *. *enterica* subsp. *arizonae* isolates, representing 46 of 102 described serovars. Nearly one-third of these are polyphyletic, including two serovars that appear in four and five distinct evolutionary lineages. Subspecies *arizonae* has a monophasic H antigen. Among the 46 serovars investigated, only 8 phase 1 H antigens were identified, demonstrating high conservation for this antigen. Prophages and plasmids were found throughout this subspecies*,* including five novel prophages. Polyphyly was also reflected in prophage content, although some clade-specific enrichment for some phages was observed. IncFII(S) was the most frequent plasmid replicon identified and was found in a quarter of *S. enterica* subsp. *arizonae* genomes. *Salmonella* pathogenicity islands (SPIs) 1 and 2 are present across all *Salmonella *, including this subspecies, although effectors *sipA*, *sptP* and *arvA* in SPI-1 and *sseG* and *ssaI* in SPI-2 appear to be lost in this lineage. SPI-20, encoding a type VI secretion system, is exclusive to this subspecies and is well maintained in all genomes sampled. A number of fimbral operons were identified, including the *sas* operon that appears to be a synapomorphy for this subspecies, while others exhibited more clade-specific patterns. This work reveals evolutionary patterns in *S. enterica* subsp. *arizonae* that make this subspecies a unique lineage within this very diverse species.

## Data Summary

All sequences analysed in this study are openly available on the National Center for Biotechnology Information (NCBI) database or Enterobase; the short-read archive accession numbers are listed in Table S1 (available in the online version of this article).

**Impact Statement**

*
Salmonella enterica* is separated into six distinct subspecies, subsp. *enterica (I)*, subsp. *salamae (II)*, subsp. *arizonae (IIIa)*, subsp. *diarizonae (IIIb)*, subsp. *houtenae (IV*) and subsp. *indica (VI*). In an era of affordable whole-genome sequencing, genomics approaches can reveal interesting phylogenetic relationships within subspecies. Given its global attribution to human and animal illness, much work has centred on subspecies *enterica*, while other subspecies have not been explored on a large scale. Subspecies *arizonae* is typically associated with reptiles and causes arizonosis in turkeys, plus a few cases of salmonellosis in humans each year. This work examined 112 subspecies *arizonae* genomes that belong to 46 different serovars and represent 45 % of known serovars of this subspecies. Whole-genome phylogeny revealed, strikingly, that nearly a third of serovars are polyphyletic, compared to the ~10 % polyphyly seen in the subspecies *enterica* serovars studied to date. Additionally, for the monophasic H antigen, we observed reduced variability, with only seven different antigens reflected in the entire subspecies. This in-depth analysis of an understudied *S. enterica* subspecies also uncovered several novel prophage elements and patterns of virulence genes among evolutionary clades. This work presents interesting findings relative to subspecies *arizonae* and builds a framework for studying other *S. enterica* subspecies.

## Introduction

*Salmonella* is divided into two species, *Salmonella enterica* and *Salmonella bongori *, with the former comprising six subspecies – *S. enterica* subsp. *enterica* (I), *salamae* (II), *arizonae* (IIIa), *diarizonae* (IIIb), *houtenae* (IV) and *indica* (VI) [1]. Subspecies can be distinguished from each other based on different biochemical tests [2]. Within these subspecies, isolates can be further classified into serovars, based on the antigenic profiles of the O and H antigens. Identical antigenic serovar profiles can occur across multiple subspecies in *Salmonella *. For example, the serovar profile 44:z4,z23:- is found in four different subspecies (*enterica*, *salamae*, *arizonae* and *houtenae*) [1].

*S. enterica* subsp. *arizonae* (subsp. *arizonae*) is the fourth largest subspecies in terms of serovar diversity, comprising 102 named serovars [1, 3–6], all of which are monophasic, harbouring only a phase 1 H antigen [1]. Diagnostically, *S. enterica* subsp. *arizonae* is distinguished from other subspecies by a series of biochemical tests, including regarding its ability to utilize malonate and liquefy gelatin, and its beta galactosidase activity, along with its inability to grow in potassium cyanide or produce γ-glutamyl transferase [2, 7]. The subspecies can also be detected by a subsp. *arizonae*-specific qPCR test that has been shown to target 56 serovars [8].

Subspecies *arizonae* is most frequently associated with reptiles, where it survives as a commensal organism, or occasionally as a pathogen resulting in septicaemia and mortality in the reptile host [4, 9–12]. However, this group has also been responsible for clinical manifestations in in a wide variety of mammals, including humans and poultry [8, 13–16]. Human infections caused by subsp. *arizonae* usually occur in immunocompromised individuals or those with underlying medical conditions [17] and are often invasive [18]. Serovars IIIa 18:z4,z23:- and IIIa 41:z4,z23:- are most often found to cause human illness and are each responsible for 10–20 cases per year [19], with the former being the third most common non-subsp. *enterica* serovar to cause human illness annually. *Salmonella* ser IIIa 18:z4,z23:- is also most commonly associated with arizonosis in turkey poults [16].

*Salmonella* genomic and phylogenetic investigations have centred mostly on diversity within subsp. *enterica*, given its size (>1500 serovars) and importance to public health and agriculture. These studies have revealed population structure and evolution within individual serovars (e.g. [19–21]) and evolutionary dynamic patterns of gene acquisition and loss, and both vertical and horizontal patterns of virulence gene inheritance across the entire subspecies [22–24]. In contrast, the phylogenetic diversity of subsp. *arizonae* is largely unknown. Molecular and genomic approaches have consistently inferred subsp. *arizonae* as sister to a large lineage containing the remaining *Salmonella* subspecies [5, 25–29]. Studies have also hinted at the presence of subsp. *arizonae-*specific loci, such as *Salmonella* pathogenicity island (SPI) 20, the *sas* fimbrial gene family, and a differential gene presence/absence in subsp. *arizonae* compared to other *S. enterica* serovars [26, 30]. Thus, the phylogenetic diversity of subsp. *arizonae* remains unknown, and the prevalence and variability of important virulence factors remains undetermined.

We performed core genome phylogenic analysis using publicly available genome data from 112 diverse *S. enterica* subsp. *arizonae* isolates to reveal the genetic structure of this subspecies. Four distinct clades were identified, and we revealed that compared to subsp. *enterica*, polyphyly within subsp. *arizonae* serovars is particularly common. The presence of some virulence genes are maintained within this subspecies, while others are highly variable.

## Methods

### Taxon sampling

We attempted to identify all public genomes available for *S. enterica* subsp. *arizonae* by querying the National Center for Biotechnology Information (NCBI) database and Enterobase [5] for isolates submitted as or determined to be ‘arizonae’ (searches performed in July 2019) (Table S1). Two datasets are presented in this manuscript: (1) a snapshot of all public *S. enterica* subsp. *arizonae* genomes we found, and (2) a trimmed phylogeny that maximizes diversity while removing clonal duplicates, and where all genomes passed minimum quality control metrics (30x coverage, 207 contigs and N50 of 10 000).

### Genome assemblies and serovar predictions

The raw sequence files for *Salmonella* genomes were downloaded from the NCBI using fastq-dump and assembly-only submissions downloaded through the NCBI’s web interface. Genomes were assembled and annotated using SPAdes (v. 3.3.1) and Prokka (v. 1.14.5), respectively [31, 32]. QUAST (v. 5.0) was used to determine the quality of the assemblies [33], and for genomes that passed this quality control, the raw reads were serotyped again internally using SeqSero2 (v1.1.1) [34]. For a small number of genomes whose serotype could not be resolved by SeqSero2, SISTR (v 1.1.1) was used [35].

### Phylogenetic inference

A multiFASTA alignment of core genes was generated from the Prokka annotation files (.gff files) using Roary (v. 3.12.0) [36] with the ‘-e MAFFT’ parameter for the alignment method. We performed this core-genome analysis with the initial capture of public genomes and again for our final, refined phylogeny. Inferring our final phylogeny was an iterative process as we determined the optimal taxon sampling to represent the diversity uncovered in this subspecies. An initial tree was inferred using FastTree (v. 2.1.10) [37] with ‘-nt’ on a single-nucleotide polymorphism (SNP) matrix derived from the Roary core gene alignment (SNPs were identified using Roary’s ‘SNP-sites’ command [38] using default parameters). This initial tree was used to refine the taxon sampling. Our trimmed final tree was inferred using RAxML (v. 8.2.9) (maximum-likelihood model GTRCAT and bootstrap replicates controlled by autoMRE) [39] on the entire core genome alignment.

### Multi-locus sequence types (MLSTs)

The seven-allele (*aroC*, *dnaN*, *hemD*, *hisD*, *purE*, *sucA* and *thrA*) MLST [40] profiles were determined using analysis results presented in Enterobase (www.enterobase.warwick.ac.uk).

### Gene analysis

Based on the comparative gene analysis among *S. enterica* subspecies by Desai *et al*. [26], we selected multiple gene families that were specific to *Salmonella* (e.g. SPI-1, SPI-2) or were specific or enriched in subsp. *arizonae*. We also selected multiple fimbrae-encoding gene families that showed differential presence across different subspecies in that paper [26]. Gene sequences were screened against subsp. *arizonae* whole genomes with blastn (v. 2.10.0+), using a cutoff of 75 % query coverage and 80 % nucleotide identity. To assess allele diversity, FASTA files containing all sequences for a single gene were generated, and the sequences were aligned using MegalignPro (DNASTAR, Lasergene v. 17) to identify and enumerate unique alleles before the Simpson’s diversity index (*D*) was calculated. CRISPR analysis was performed using CRISPRCasFinder [41, 42].

### Prophage identification

PHASTER [43] was used to identify intact phage genomes. Prophages characterized as matching known phage sequences were recorded, using a 50 % threshold of coding sequences (CDSs), according to the output from PHASTER. For prophages detected by PHASTER but not identified (i.e. had <50 % CDS matches with a known phage), the prophage genomes were annotated using GeneMark [GeneMark with Heuristic models for prokaryotes (version 3.25)] [44]. blastp was used to compare these sequences to each other to identify similar prophages with 75 % identity [45]. For groups of similar prophages, a representative prophage that was present in the middle of a contig, and had the largest number of CDSs, was used to compare to other phages in that group. Phages were scored as the same if they shared at least 50 % CDSs.

### Plasmid analysis

Plasmid Finder 2.0 [46] was used to identify *Enterobacteriaceae* plasmid replicons within our assembled genomes with a 95 % minimum identity threshold and 60 % minimum coverage.

## Results

### Overview of sample dataset

A total of 334 *S. enterica* subsp. *arizonae* genomes and outgroups were downloaded from our initial query of public data (Table S1). Our initial Roary analysis produced a core genome of 2546 genes and 110 SNPs, which was used to infer the initial tree using FastTree (Fig. S1). From this large tree we selected 111 subsp. *arizonae* genomes that maximized genomic, serovar and collection source diversity, retaining multiple genomes from each serovar where possible (Table S1). In trimming the tree, we retained all serovars that were in the dataset, and for serovars with only one or two representative genomes, all available genomes with good assemblies were kept. For serovars that were abundant in the dataset, such as IIIa 18:z4,z23:- (61 genomes) or IIIa 41:z4,z23:- (96 genomes), we kept a smaller subset of genomes. For serovar IIIa 18:z4,z23:-, 59 genomes were from isolates collected from ground turkey, and 2 were from humans. We kept both human isolates and four representative ground turkey isolates in our final analyses. For serovar IIIa 41:z4,z23:-, 15 genomes were retained and these were selected to retain source diversity [human (6), environment (1), turkey (1), chicken (1) fresh produce/vegetables (1), reptiles (2), companion animal (1) and unknown (2)]. Within large source groups (e.g. 67 genomes for IIIa 41:z4,z23:- from human isolates), we further selected based on phylogeny to capture genetic diversity within the serovar. We also added the genome for ATCC strain BAA-731 (NC_010067) that was available as a closed genome on the NCBI database. This final set of 112 subsp. *arizonae* genomes included isolates from the USA (54 %), the UK (21 %) and Mexico (13 %), plus others. A total of 35 % were isolated from humans, 15 % from plants or produce, 15 % from poultry sources and only 3.6 % from reptiles. The trimming process to remove several genomes from our final analyses also limited biases associated with source attribution. In the example above for serovar IIIa 18:z4,z23:-, retaining all 61 genomes in our analyses would have suggested that nearly a fifth of subsp. *arizonae* come from ground turkey. There were six genomes retained in our final dataset where SeqSero could not resolve the O antigen. SISTR was able to resolve four of these (Table S1). The remaining two genomes [from strains, FDA00002278 (z29:-) and FMA0016 (z36:-)] were not included in the sum of different serovars, as these H antigens occur in multiple subsp. *arizonae* serovars. A total of 46 of the 102 subsp. *arizonae* serovars were represented in our dataset [1, 3], and for 24 of these, between 2 and 15 genomes were used. We also included 14 outgroups representing the other 5 *S. enterica* subspecies, plus *S. bongori *, bringing the final dataset total to 126 *Salmonella* genomes. Since subsp. *enterica* has been well studied and is the largest subspecies, we included more outgroups from this subspecies and represented the major evolutionary clades of this subspecies [24]. The average genome size for the group of 126 genomes was 4.6 Mb (Table 1).

**Table 1.** Genome assembly statistics

	Average	Standard deviation	Minimum	Maximum
Genome size (Mb)	4.6	0.16	4.4	5.71
Number of contigs	69.5	29.7	1	207
N50 (Kb)	217	94	55.3	605

**Fig. 1.**
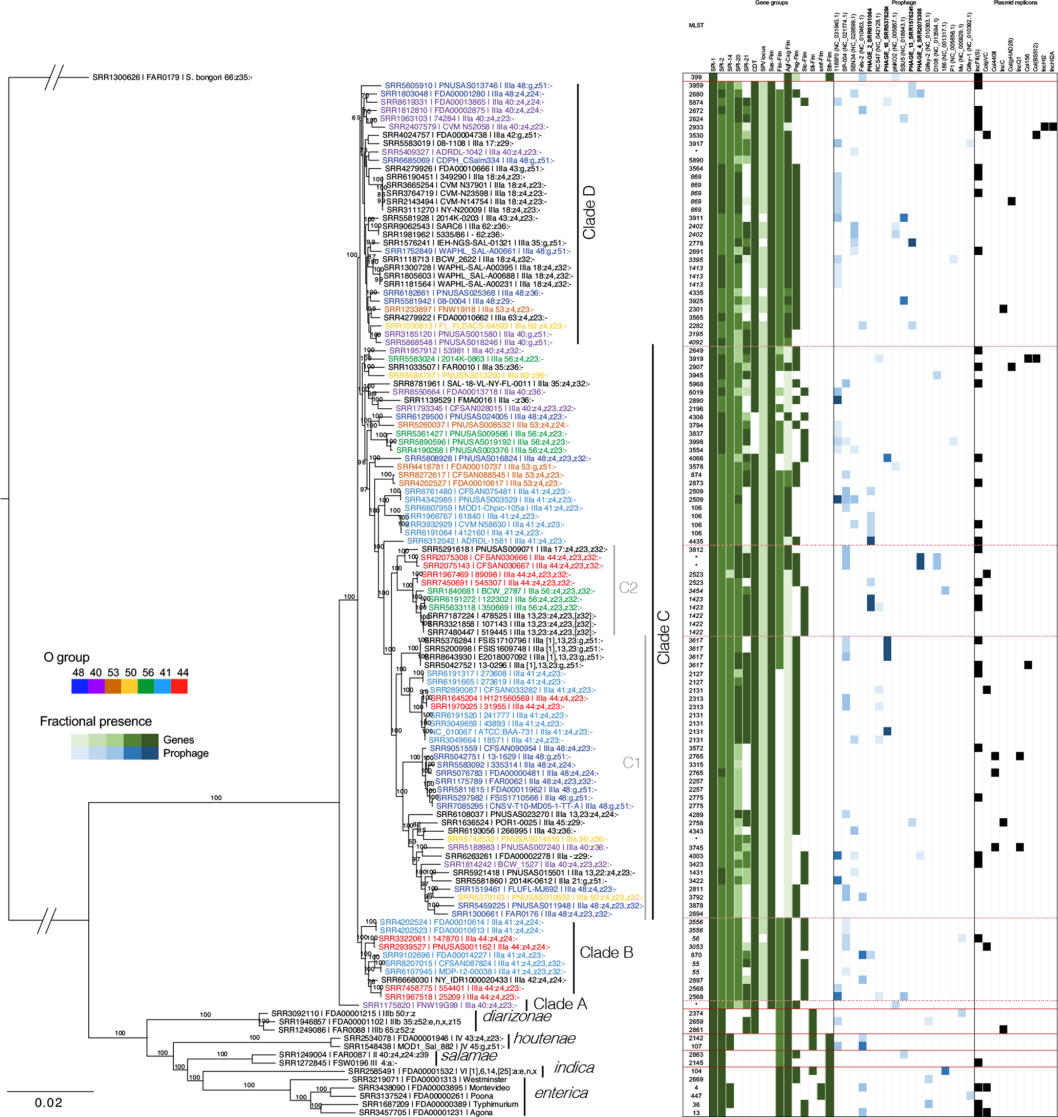
*
S. enterica* subsp. *arizonae* phylogeny, distribution of mobile genetic elements and virulence genes. Maximum-likelihood phylogeny of *Salmonella* inferred with RAxML from a core genome alignment with the named clades indicated, and separated by dashed red lines on the heat map. Polyphyletic serovars are shown in different colours; since there are a large number of polyphyletic serovars, they are separated by O group to provide some clarity. The italicized MLST profiles are from monophyletic serovars. The percentage of genes within a particular gene family is shown, according to the legend. Gene families with <50 % of the genes are not shown, and the individual gene analysis is listed in Table S3. Conservation of prophage sequences are shown based on an ‘intact’ score from PHASTER and presence in three or more genomes. Only prophages with 50 % or more matching CDSs are shown, according to the scale in the legend, with the exception of phage 4, where a number of matching phages were located at the end of a contig and are represented in grey. Raw data for phage are shown in Table S4. Plasmid replicon presence is indicated by black boxes.

Our final Roary analysis on the trimmed dataset produced a core genome comprising 2512 genes across 2 470 851 nucleotides. *S. enterica* subsp. *arizonae* is monophyletic, forming a lineage sister to the other five *S. enterica* subspecies (Fig. 1). We identified four clades within subsp. *arizonae*, A–D, based on their distribution within the tree and identification of four major splits at the base of the tree. Clades A, B and C were strongly supported, each having 100 % bootstrap support (BS), while clade D had moderate 69 % BS. Clade B is sister to C and D, and clade A is sister to clades B–D. Clade A is represented by a single isolate (FNW19G99), which diverged earlier than the other *S. enterica* subsp. *arizonae *. Clade C is the largest clade with 69 genomes, and contains two major subclades, C1 and C2.

### High levels of polyphyly in subspecies *arizonae*

Among *S. enterica* subsp. *arizonae *, 17 different O antigens and 7 different phase 1 H antigens were present. Nearly one-third of the serovars (14/46; 30 %) are polyphyletic, with five serovars having three or more lineages (Table 2). Serovar IIIa 41:z4,z23:- is present in five distinct lineages across clades B and C (Fig. 1). One of these lineages is also paraphyletic and is separated by a single lineage of serovar, IIIa 44:z4,z23:-, that is nested within it. Serovar IIIa 48:g,z51:- is also highly polyphyletic, being separated into four different lineages across clades A and B. There were four polyphyletic serovars for whom all lineages resided in clade C: IIIa 48:z4,z23:- (three lineages), IIIa 48:z4,z23,z32:- (three), IIIa 50:z36:- (two) and IIIa 56:z4,z32:- (two).

**Table 2.** Polyphyletic subspecies *arizonae* serovars

			No. of genomes per lineage			
Serovar	# lineages	Lineage 1	Lineage 2	Lineage 3	Lineage 4	Lineage 5
IIIa 41:z4,z23:	5	6	5*	2	1	1
IIIa 48:g,z51:	4	4*	1	1	1	
IIIa 40:z4,z23:-	3	2	1	1		
IIIa 48:z4,z23,z32:-	3	2	1	1		
IIIa 48:z4,z23:-	3	1	1	1		
IIIa 40:z36:-	2	1	1			
IIIa 40:z4,z23,z32:-	2	1	1			
IIIa 40:z4,z24:-	2	1	1			
IIIa 44:z4,z23,z32:-	2	2	2			
IIIa 44:z4,z23:-	2	2	2			
IIIa 48:z4,z24:-	2	2	1			
IIIa 50:z36:-	2	1	1			
IIIa 53:z4,z23:-	2	2	1			
IIIa 56:z4,z23:-	2	2	1			

*These lineages are distinct from other lineages within the serovar and are also paraphyletic.

We identified 96 MLSTs in total, 84 of which were for subsp. *arizonae* genomes. ST869 and ST2131 were the most common MLSTs, and each corresponded to five genomes (Fig. 1). In general, the MLST diversity matched the phylogenetic analysis; specifically, polyphyletic lineages were reflected by the MLST profile. The allelic profiles for each MLST show, expectedly, that closely related genomes differ by a small number of alleles (Table S2). There were 10 monophyletic serovars represented by multiple genomes and only 4 of these could be separated by MLST (IIIa 18:z4,z32:-, IIIa 40:g,z,51:-, IIIa 44:z4z24:- and IIIa 56:z4,z23,z32), demonstrating the increased discrimination provided by whole-genome sequencing. No genomes belonging to different serovars shared an MLST profile.

### Distribution of *Salmonella* pathogenicity islands (SPIs), virulence genes and fimbrial operons

**Fig. 2.**
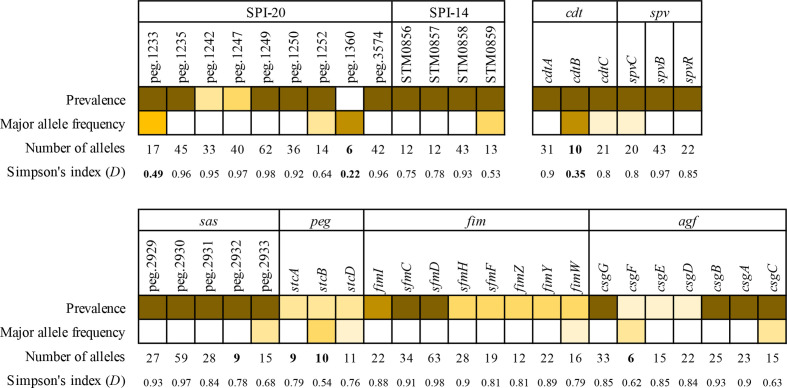
Allelic diversity among loci conserved in subspecies *arizonae.* The relative prevalence of each gene across subsp. *arizonae* and the relative frequency of the major allele of each gene are both depicted as a heat map according to the legend shown. Genes with 10 or fewer alleles are highlighted in bold, as are genes with a *D* <0.5. Loci selected for this analysis were either present in the majority of subsp. *arizonae* genomes and enriched in subsp. *arizonae* compared to the outgroups (SPI-14, SPI-20, *cdt, spv*, *sas, peg*) or showed some interesting clade-specific patterns (*fim, agf*). Genes labelled with ‘peg’ identifiers are from [26] (523833.3), and descriptions of these genes are provided in able S3.

Like all *S. enterica* subspecies, subsp. *arizonae* also contains SPIs 1 and 2 (Fig. 1, Table 3). Interestingly, the SPI-1-secreted effector-encoding genes, *sipA*, *sptP* and *arvA*, are missing in all subsp. *arizonae* genomes (Table S3). Similarly, the SPI-2 effector-encoding genes *sseG* and *ssaI* are absent from all subsp. *arizonae* isolates, and *ssaO* was missing from all but seven. Our data show that SPI-20 is present and well maintained throughout subsp. *arizonae* (Fig. 1, Table 3). The *vgrG* gene (peg.1360) in SPI-20 is missing from clade B (Table S3), but is highly conserved among other subsp. *arizonae* genomes, with a low allele frequency and low Simpson’s diversity index (*D=*0.22; Fig. 2). The SPI-21 locus is less well maintained and is missing entirely in 16 subsp. *arizonae* genomes (Tables 3 and S3), including a small group in subclade C1 that consists of 4 different serovars all sharing the O 48 antigen. SPI-21 is also absent in one of the three subsp. *diarizonae* genomes. We examined two other virulence loci that have previously been found in subsp. *arizonae*, *spv* (* Salmonella* plasmid virulence) and *cdt* (cytolethal distending toxin). The *spv* locus is present in 95 % of all subsp. *arizonae* genomes, including all in clade C. The *cdt* locus was conserved in subsp. *arizonae* and only missing from a small lineage of five genomes. Compared to other genes at the *cdt* locus, *cdtB* was particularly well conserved, with only 10 unique alleles and a *D* value of 0.35 (Fig. 2).

**Table 3.** Presence of pathogenicity islands, virulence genes and *fim* operons in subspecies *arizonae* clades B–D*

Clade (no. of genomes)	SPI-1	SPI-2	SPI-14	SPI-20	SPI-21	*Cdt*	*Spv*†	*Sas*	*Peg*	*Fim*	*Agf/csg*	*Stc*
Clade B (10)	100 %	100 %	100 %	100 %	40 %	100 %	90 %	100 %	0 %	100 %	100 %	90 %
Clade C (69)	100 %	100 %	99 %	97 %	51 %	93 %	100 %	100 %	61 %	46 %	34 %	23 %
Clade D (32)	100 %	100 %	100 %	100 %	38 %	100 %	88 %	100 %	66 %	97 %	97 %	3 %

*For a genome to be included in this analysis, 80 % of the genes in an operon had to be present.

†The spv locus contains four genes, and only two of these are found in subspecies *arizonae*, so for this locus, the threshold was set to 50 % of the genes (i.e. two genes).

*S. enterica* encodes a number of fimbrial operons, whose presence varies considerably among the different subspecies. Desai *et al.* demonstrated that a number of these were enriched or exclusive to subsp. *arizonae* [26]. We interrogated eight of these further to determine how broadly they were found in subsp. *arizonae*. Our dataset shows that the *sas* operon and its five genes are exclusive to subsp. *arizonae*, though we note the small number of outgroup genomes considered here (Fig. 1, Table S3). The *fim* operon is present in all subsp. *arizonae* genomes except those in subclade C1. The *agf/csg* operon is also widely distributed across subsp. arizonae, but less well maintained (<60 % inclusion of all genes in the operon) in both C1 and C2 subclades. The *peg* and *stc* fimbral operons are found throughout subsp. *arizonae*, although *peg* has been lost from clade B, while *stc* is enriched in clade B (Table 3). The average number of alleles for each fimbrial gene was 23, with high diversity (average *D*=0.82)(Fig. 2). While the number of alleles for the three genes in the *peg* locus is low compared to other loci, this is likely due to the absence of this locus in many subsp. *arizonae* genomes, as the concurrent *D* value is high (Fig. 2). The remaining fimbral operons we investigated, *sth, sfi* and *smf*, are all absent from subsp. *arizonae*, with only a single gene in the *sfi* operon found in 47 genomes (Table S3).

### Distribution of mobile genetic elements and identification of novel prophage

The presence of mobile genetic elements such as phage and plasmids can reveal interesting phylogenic patterns. PHASTER identified 212 ‘intact’ prophages among the 126 genomes, including 177 within subsp. *arizonae* genomes. For prophage detected by PHASTER but not identified (<50 % CDS matches), prophage sequences were aligned to each other to identify related phages (Table S4). In total, there were 17 different prophage sequences that were present in at least 3 genomes (Fig. 1). One isolate (PNUSAS003529) contained 7 intact phages, and 23 genomes were devoid of intact prophages (Fig. 1). Four common *Salmonella* phages, 118 970, SP-004, SEN34 and Fels-2, were found in subsp. *arizonae* genomes (Fig. 1). We note some phylogenetic differences in phage distribution: SEN34 was identified in 13 isolates across clades C and D. SP-004 was only found in clades B and C, and entirely absent from clade A. Gifsy-1 and Gifsy-2 are *Salmonella* phages that are abundant throughout subsp. *enterica* [24]; Gifsy-2 was absent from all subsp. *arizonae* genomes, and Gifsy-1 was only found in a single subsp. *arizonae* genome and with a low percentage identity (not shown in the figure). We identified seven novel phages in this study (Se_ariz_Phage 1–7) that were all present in subsp. *arizonae* and did not match to any phages present on the NCBI database. Phage 1 and phage 6 were also present in one subsp. *enterica* isolate (serovar Westminster). Phages 2 and 4 were the most abundant and enriched in clade C, while phage 5 and phage 7 were absent from clade C. Given that clade A was formed by a single genome, the phage diversity in this clade cannot be assessed. In general, polyphyletic serovars showed different prophage patterns in the different lineages. For example, across the 15 genomes of serovar IIIa 41:z4,z23:-, there were 7 different prophages, reflecting the diversity of this serovar

We only scored intact prophages for this analysis; prophages separated over two or more contigs, which occurs often as the repetitive elements in phage genomes can be challenging to assemble, would likely not be scored as intact according to PHASTER. Therefore, it is likely that some of these subsp. *arizonae* phages may be distributed more broadly, but were missed by our analysis. For example, there are 14 prophages that share very high similarity (Table S4) and despite being scored as ‘intact’, many of them are located at the end of a contig. On further examination, these phages match well to phage 4, and this match is reflected in Fig. 1 (grey boxes).

A total of 51 plasmid replicons were identified, representing 10 different replicons (Fig. 1), with a maximum of 3 replicons found in 1 genome assembly. IncFII(S) was the most common plasmid replicon and was found in 29 subsp. *arizonae* genomes plus other *S. enterica* subspecies and *S. bongori *. The next most frequently identified plasmid replicon was ColpVC. Both IncFII(S) and ColpVC replicons were broadly distributed across subsp. *arizonae* clades B–D. Overall, the presence of mobile genetic elements does not appear to follow any clade-specific trend. Unlike other *S. enterica* subsp., we found no evidence of CRISPR arrays within subsp. *arizonae.*

## Discussion

*S. enterica* subsp. *arizonae* is infrequently associated with human illness, but when it is attributed to illness, it is significantly more likely to cause invasive extraintestinal disease [18]. In this study we examined the phylogenetic relationships between a broad collection of 112 subsp. *arizonae* genomes, and found that they formed 4 major clades, 3 of which had strong bootstrap support. Although we took an unbiased snapshot of available data for this subspecies, we recognize potential bias in two areas. First, even though subsp. *arizonae* is commonly associated with reptiles [12, 47–49], there is an over-representation of available genomes from human origin, or from food, reflecting the public health surveillance focus. Second, most sequences are from the USA and the UK, reflecting their sequencing efforts and submission to public databases. Nonetheless, with this study we have been able to interrogate the phylogenetic relationships among 45 % of *S. enterica* subsp. *arizonae* serovars.

### Increased polyphyly in *S. enterica* subsp. *arizonae*

Serovars are generally thought of as natural groups, but whole-genome sequencing and other molecular approaches have demonstrated that polyphyly exists within multiple serovars [23, 24, 50, 51]. For example, serovar Newport has three genetically distinct lineages [52] and serovar Kentucky has two well-defined lineages [53, 54]. MLST analysis previously demonstrated that up to 50 % of *S. enterica* subsp. *enterica* serovars were polyphyletic, though this approach relied on sequence information from only seven loci [55]. Most recently, Worley and colleagues used whole-genome sequence data and showed that 9 % of ~250 subsp. *enterica* serovars (representing 17 % of subsp. *enterica* serovars) were polyphyletic [24]. Evolutionarily, recombination between *Salmonella* occurs frequently and, again, most studies have been performed in subsp. *enterica* [27, 51, 56–59]. Here, in our investigation of 45 % of subsp. *arizonae* serovars, we show that 30 % of the serovars are polyphyletic, significantly higher than is observed for subsp. *enterica*. Reptiles are considered to be a natural reservoir of subsp. *arizonae* [4, 9–12], and since some strains of this subspecies are considered commensal in reptiles, they are more likely to harbour multiple different strains at a given time. This could potentially facilitate more recombination events and the subsequent generation of polyphyletic serovars. The high incidence of polyphyly observed in subsp. *arizonae* suggests that recombination is frequent in this subspecies. Two serovars, IIIa 41:z4,z23:-, which is moderately associated with human illness, and serovar IIIa 48:g,z51:-, are highly polyphyletic, with five and four distinct lineages, respectively. Though unlikely given the concordance between the reported and predicted serovar, it should be noted that a single genome switching serovar may cause both serovars to be labelled as polyphyletic. It remains to be determined whether other *S. enterica* subspecies have similar levels of polyphyly, as studies to date have centred on subsp. *enterica* and now here on subsp. *arizonae.* Similar evolutionary studies using large numbers of isolates will resolve this for other subspecies and provide a picture of polyphyly across the entire *S. enterica* species. Nonetheless, this work continues to underscore the importance of characterizing *Salmonella* isolates by whole-genome sequencing or molecular typing as polyphyly remains hidden when only serotyping is used to characterize *Salmonella *.

### Reduced variability of H antigen identity in *S. enterica* subsp. *arizonae*

The H antigen in subsp. *arizonae* is monophasic. Interestingly, there is low variability in the phase 1 H antigen across subsp. *arizonae*, and we only found seven different phase 1 antigens (z29, z36, g,z51, z4,z23, z4,z23,z32, z4,z24, z4,z32), with a quarter (11/46) having the z4,z23 phase 1 antigen. Examination of the Kaufman–White *Salmonella* serotyping scheme [1, 3, 6] shows that these 7 H antigens are the only ones found throughout the subspecies, with 22 of the 102 serovars having the z4,z23:- antigen and 18 having the g,z51:- antigen. Subspecies *enterica* serovars can colonize a wide variety of animal hosts and a survey of the top 32 subsp. *enterica* serovars associated with human illness [60] showed that even within this small group there are 20 different phase 1 H antigens. Thus, compared to subsp. *enterica*, the low phase 1 H antigen variability in subsp. *arizonae* is unusual and we postulate that this may be the result of the limited host range of this subspecies, whose members are most frequently associated with reptiles and turkeys [4, 9, 10, 12, 16]. Interestingly, we note that when the seven phase 1 H antigens in subsp. *arizonae* are also found in other subspecies, they are commonly associated with serovars that also harbour monophasic H antigens. For example, the z4,z24 antigen is found in 26 subsp. *enterica* serovars and 14 subsp. *salamae* serovars, and the H antigen is monophasic in 23 and 10 of these, respectively [1].

### Prophage distribution among *S. enterica* subsp. *arizonae* lineages

The presence of prophages within bacterial genomes contributes to horizontal gene transfer in *Salmonella* [61, 62]*,* and prophage content, even within a single serovar, can be diverse [63]. Many prophages that are broadly found across other *S. enterica* subspecies, such as Fels-2, 118 970 and SEN34, are also abundant in subsp. *arizonae.* Conversely, other temperate phages typically enriched in subsp. *enterica,* such as Gifsy-1, are largely absent from subsp. *arizonae.* The complete absence of Gifsy-2 suggests that this prophage was acquired by other *Salmonellae* after divergence of subsp. *arizonae.* Prophage are distributed broadly throughout subsp. *arizonae*, suggesting that there has been frequent phage transmission among subsp. *arizonae* individuals. Further, there were no significant lineages that lacked prophages. The increased number of subsp. *arizonae* genomes that have been interrogated here enabled the identification of seven novel prophage genomes, most of which seem specific for this subspecies, and which are also distributed broadly among the genomes investigated. Plasmid replicon presence was also diverse, with no clear evolutionary pattern to the presence of a particular replicon. Similar to phage distribution, this suggests easy plasmid transmission within subsp. *arizonae* and with other *Enterobacteriaceae *. Plasmids with IncII replicons are commonly found in *Salmonella *, and IncII(S) was the most abundant replicon identified in this study.

### Evolution of SPIs in *S. enterica* subsp. *arizonae*

Function of SPI-1 and SPI-2 type III secretion systems and their effectors have been well characterized in *S. enterica* subsp. *enterica* serovar Typhimurium. The former has an important function in gastrointestinal illness, and the latter functions in both gastrointestinal illness and systemic infections [64–67]. SPI-2 was acquired by *S. enterica* after divergence from *S. bongori *, but before separation into subspecies, and this may contribute to the broad host range of *S. enterica* [68]. Variability in SPI-2 effector content across subsp. *arizonae* has been described previously by microarray analyses of five isolates [29]. Notably, the absence of *sseG*, a core SPI-2 effector [69, 70], in subsp. *arizonae,* may affect the ability of subsp. *arizonae* to cause illness. Previous publications identified the type VI secretion system encoding SPI-20 as being exclusive to subsp. *arizonae* and that SPI-21, also encoding a type VI secretion system, was only found in subsp. *arizonae* and *diarizonae* [26, 30]. By examining a large number of genomes, we have been able to confirm the presence and maintenance of SPI-20 in subsp. *arizonae*, and also show that SPI-21 is absent from a number of subsp. *arizonae* genomes, and also in one of the three supbspecies *diarizonae* genomes in our sample set.

We observed that two pathogenicity loci, *spv* and *cdt*, were enriched in subsp. *arizonae*, with the latter also being found in the three subsp. *diarizonae* genomes we included in this study. In concordance with another study we also find that *spvD*, which encodes a cysteine hydrolase, is absent from all subsp. *arizonae* genomes analysed here [47, 71]. While *spv* was not identified in our outgroup subspecies, it has been documented to occur on plasmids found in many subsp. *enterica* serovars, where it is exclusively present on a plasmid [72]. This locus is absent in subsp. *diarizonae* and subsp. *indica*, as we also observe here [73]. Further, there is evidence that the *spv* locus has been transferred from subsp. *enteric* to subsp. *arizonae* [73].

Fimbrial-mediated adhesion precedes cellular invasion, and repertoires of fimbrial adhesions drive attachment to different host target cells [74]. We examined *Salmonella* fimbrial operons, finding that the *sas* operon was likely synamorphic for *S. enterica* subsp. arizonae*,* and that the *fim*, *agf*, *peg* and *stc* operons were found in multiple *S. enterica* lineages. Operons *sth, sfi* and *smf* were all absent in *S. enterica* subsp. arizonae*.* Given fimbrial protein function, this differential gene presence may guide the colonization of different hosts among different *Salmonella* lineages. While we observed conservation of several operons, evidence of low allelic diversity was lacking, which may speak to differential gene activity, although this warrants further investigation.

In sum, phylogenetic studies can reveal much about the evolutionary history and biology of this important pathogen and, to date, studies have focused on *S. enterica* subsp. *enterica* [23, 24, 55] due to its clinical relevance, but also on other salmonellae such as *S. bongori* [75]. The current study presents the first in depth phylogenetic analyses of *S. enterica* subsp. *arizonae *. While evolutionary parallels can be drawn with subsp. *enterica*, there are distinct differences, including increased polyphyly and low H antigen varibility. Future phylogenetic studies investigating the remaining four *S. enterica* subspecies will continue to shed light on *Salmonella* evolution.

## Supplementary Data

Supplementary material 1
